# Bisphenol S negatively affects the meotic maturation of pig oocytes

**DOI:** 10.1038/s41598-017-00570-5

**Published:** 2017-03-28

**Authors:** Tereza Žalmanová, Kristýna Hošková, Jan Nevoral, Kateřina Adámková, Tomáš Kott, Miloslav Šulc, Zora Kotíková, Šárka Prokešová, František Jílek, Milena Králíčková, Jaroslav Petr

**Affiliations:** 1 Czech University of Life Sciences Prague, Faculty of Agrobiology, Food and Natural Resources, Department of Veterinary Sciences, Prague, Czech Republic; 2 Charles University in Prague, Faculty of Medicine in Pilsen, Biomedical Center and Department of Histology and Embryology, Pilsen, Czech Republic; 3 Institute of Animal Science, Prague, Czech Republic; 4 Czech University of Life Sciences Prague, Faculty of Agrobiology, Food and Natural Resources, Department of Chemistry, Prague, Czech Republic

## Abstract

Bisphenol A (BPA), a chemical component of plastics, is a widely distributed environmental pollutant and contaminant of water, air, and food that negatively impacts human health. Concerns regarding BPA have led to the use of BPA-free alternatives, one of which is bisphenol S (BPS). However, the effects of BPS are not well characterized, and its specific effects on reproduction and fertility remain unknown. It is therefore necessary to evaluate any effects of BPS on mammalian oocytes. The present study is the first to demonstrate the markedly negative effects of BPS on pig oocyte maturation *in vitro*, even at doses lower than those humans are exposed to in the environment. Our results demonstrate (1) an effect of BPS on the course of the meiotic cell cycle; (2) the failure of tubulin fibre formation, which controls proper chromosome movement; (3) changes in the supply of maternal mRNA; (4) changes in the protein amounts and distribution of oestrogen receptors α and β and of aromatase; and (5) disrupted cumulus cell expansion. Thus, these results confirm that BPS is an example of regrettable substitution because this substance exerts similar or even worse negative effects than those of the material it replaced.

## Introduction

Many anthropogenic substances introduced to the environment exert endocrine-disrupting effects and negatively affect animal and human health by altering the functions of various endogenous hormones, even at very low doses^1^. Because reproduction is subject to complex endocrine regulation, the effects of low-dose endocrine disruptors may severely impact reproductive processes. Bisphenol A (BPA) is a known endocrine disruptor and a component of most plastics, allowing it to reach not only the domestic environment but also water and food supplies^2^. In addition to affecting many other physiological processes^3^, BPA may significantly affect reproductive physiology^4–6^. Low-dose exposure to BPA during prenatal and neonatal development has been linked to a wide variety of effects, including alterations in brain sexual differentiation, male and female reproductive tract defects, pregnancy complications, and meiotic abnormalities in foetal oocytes^7, 8^. Oestrogenic properties of BPA are known as one of known molecular action in reproductive system^9^. For these reasons, the use of BPA was restricted, and a number of products are sold with the guarantee that they are BPA-free.

In BPA-free products, the forbidden BPA has been replaced by other substances, of which the most widely used is bisphenol S (BPS)^10^. BPS is used compound in common plastics, canned items, receipt papers and many others^8^. Therefore, global production of BPS is rising sharply^11^. Massive exposure to BPS has been observed in many populations worldwide^12^. BPS simulates the actions of oestrogens, and a number of studies have demonstrated the negative effects of BPS on a wide range of physiological processes^13^. There are many indications that BPS has become a “regrettable substitution”, specifically, that the endocrine disruptor BPA has been replaced by a substance that exerts vigorous endocrine-disruptive effects^14, 15^. A recent examination of urine samples in the United States and Asia confirmed previous work showing that 93% of people had detectable levels of BPA but surprisingly showed that 81% had detectable levels of BPS^16^. Moreover, BPS has been detected in human blood serum^17^. Thus, its possible effects on highly sensitive physiological functions, such as reproduction, must be elucidated. Meiotic maturation of oocytes is a highly sensitive reproductive physiological process. The presence of BPS in body fluids prompts the question of whether BPS exposure disrupts oocyte maturation. Given this, it is troubling that information regarding the influence of BPS on mammalian oocytes remains lacking.

The process by which mammalian oocytes form a complex with surrounding cumulus cells to prepare for fertilization is dependent on hormonal stimuli. Utilizing stored RNA and stored and newly synthesized proteins, oocytes undergo a complex series of processes termed meiotic maturation^18^, which includes breakdown of the nuclear membrane and chromatin condensation (germinal vesicle (GV) breakdown), as well as the formation of microtubule-organizing centres for spindle division. Chromosomal movement is necessary for meiosis I and meiosis II and for extrusion of the polar body^19^. Flawless tubulin function during these processes is required for the meiotic cell cycle to proceed successfully^20^. Oestrogens and aromatase regulate the maturation of mammalian oocytes, which plays a crucial role in steroidogenesis^21^. Therefore, the influence of BPS on oestrogen receptors and aromatase demands attention. Cumulus cells also respond to disrupted hormonal signalling by altering the production of hyaluronic acid (HA), the most abundant compound of their extracellular matrix^22^.

We selected pig oocytes as a suitable model to study endocrine disruption in mammalian oocytes. The maturation time of a pig oocyte is longer than that of the commonly used mouse model, therefore providing an opportunity to carry out a more detailed study of the cell cycle. Pig oocytes are also larger in size, permitting the more detailed evaluation of phenomena related to tubulin alterations resulting from greater functional distances between the meiotic spindle and chromosomes, which the microtubules must span. In addition, pig oocytes are physiologically more similar to human oocytes than mouse oocytes, and thus our results provide a more solid basis for human reproductive research^23^.

The aim of this study was to explore the effects of BPS on the *in vitro* maturation of porcine oocytes. The results reported here are the first to demonstrate the detrimental effects of BPS on the maturation of mammalian oocytes *in vitro*, indicating the regrettable substitution of BPS for BPA merits our attention with respect to mammalian reproduction.

## Results

Our analysis of pig follicular fluid confirmed the absence of BPS. On the basis of this observation (see Supplementary Tables S1 and S2), we suspected that cumulus-oocyte complexes were not influenced by BPS before isolation. Moreover, the viability of oocyte and cumulus cells was tested. After 24 and 48 h of *in vitro* culture, none of the BPS treatments (3 nM, 300 nM or 30 µM) influenced the viability of oocytes and cumulus cells (see Supplementary Table S3A and S3B).

### Both progression to MI and MII were sensitive to BPS

**Figure 1:**
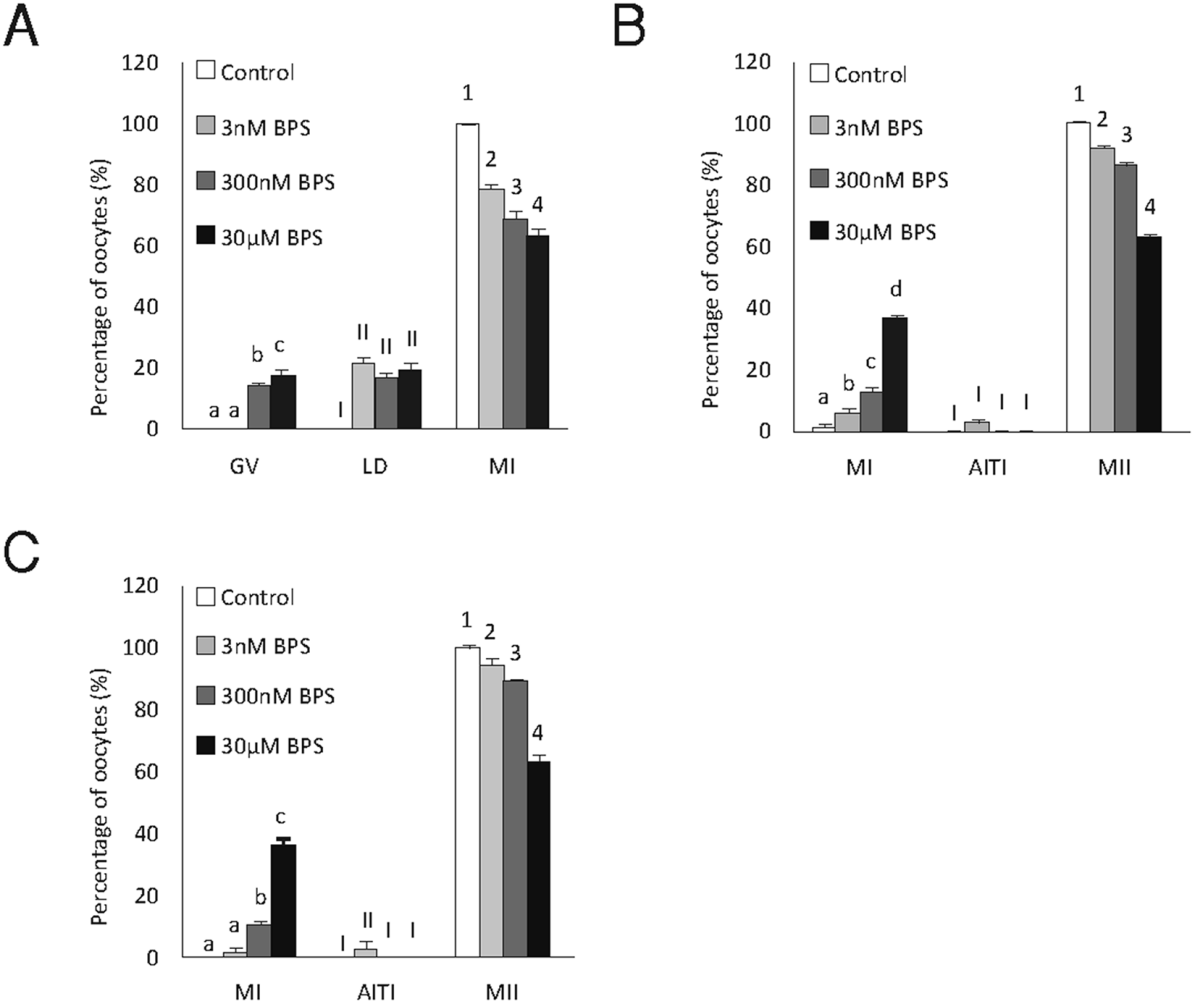
Effects of BPS on the meiotic maturation of oocytes. Effects of BPS (3 nM, 300 nM, and 30 μM) on the stages of meiotic maturation achieved by oocytes cultured for (**A**) 24 h, (**B**) 48 h, and (**C**) 72 h *in vitro*. GV – germinal vesicle, LD – late diakinesis, MI – metaphase I, AITI – anaphase I–telophase I, MII – metaphase II. The data are expressed as the mean ± SEM from four independent experiments, n = 120 oocytes per group. Different superscripts denote the statistical significance among experimental groups within the same stage of meiotic maturation (P < 0.05).

Under *in vitro* conditions, pig oocytes mature to metaphase I (MI) after 24 h and to metaphase II (MII) after 48 h. Cumulus–oocyte complexes (COCs) treated with various concentrations of BPS (3 nM, 300 nM or 30 µM) exhibited a significant dose-dependent decrease in MI and MII stage achievement after 24 and 48 h of *in vitro* culture. BPS-treated oocytes (300 nM and 30 µM) did not resume meiosis after 24 h of *in vitro* culture. However, after 48 h of *in vitro* culture, all BPS-treated oocytes initiated meiotic maturation and matured to at least MI (Fig. 1A,B).

After 72 h of culture (Fig. 1C), maturation was not only delayed but also disrupted and blocked by BPS in all used concentrations. This meiotic block was irreversible because maturation did not improve even after 48 h of culture with BPS followed by culture in a BPS-free medium (see Supplementary Fig. S1A). Both progression to MI and to MII are sensitive to all tested concentration of BPS: maturation decreased in COCs exposed to BPS for the first 24 h (Fig. S1B) or during the second 24 h of 48 h of overall culture in dose dependent manner (Fig. S1C).

### α-tubulin assembly during porcine oocyte maturation after BPS treatment

**Figure 2:**
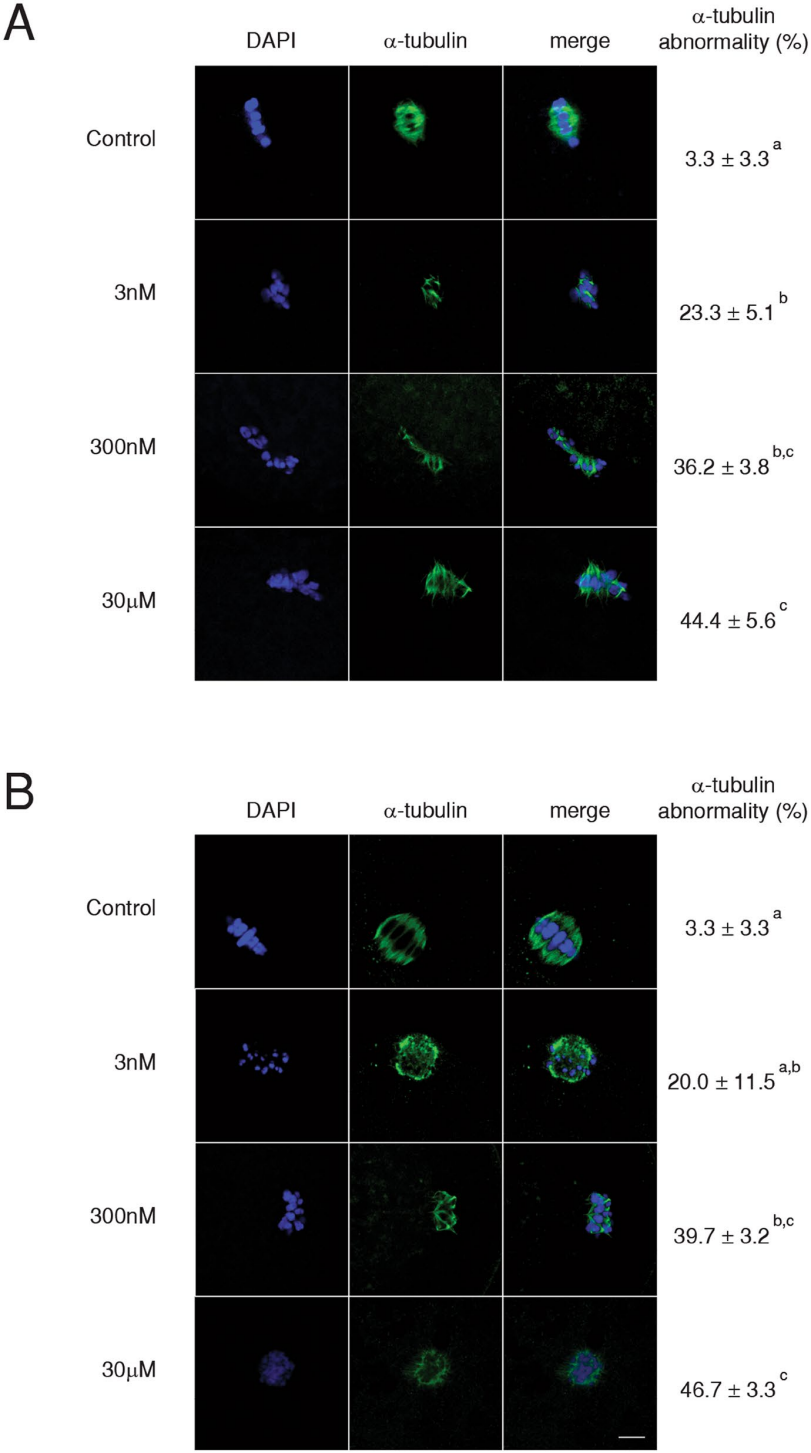
Effects of BPS on meiotic spindle formation during the maturation of porcine oocytes. Representative pictures showing defects in the morphology of spindle organization and chromosome alignment in oocytes after 24 h (**A**) or 48 h (**B**) of culture *in vitro* after BPS (3 nM, 300 nM, and 30 μM) treatment. Green colour indicates α-tubulin, blue indicates DAPI. Scale bar = 10 µm. Percentage of α-tubulin abnormalities after 24 h (n = 83) and 48 h (n = 82) of culture *in vitro* are presented to the right side of the images. The data are expressed as the mean ± SEM of three independent experiments. Different superscript letters denote statistical significance (P < 0.05).

Faultless organization of tubulin filaments and chromosomes in the spindle apparatus is required for correct meiotic maturation to be achieved. We observed several types of defects, including swollen chromosomes and irregular organization, decreased numbers of tubulin filaments, spindles in a circular formation or astral arrangement, elongated metaphase plates, and reduced spindle size. These phenomena were apparent in both MI (Fig. 2A) and MII (Fig. 2B) oocytes and were present even in the 3 nM BPS treatment group after 24 and 48 h of *in vitro* culture, respectively. BPS dramatically affects the formation and structure of the meiotic spindle (see Supplementary Video 1).

### Effects of BPS on the amount of mRNA for oestrogen receptors and aromatase

**Figure 3:**
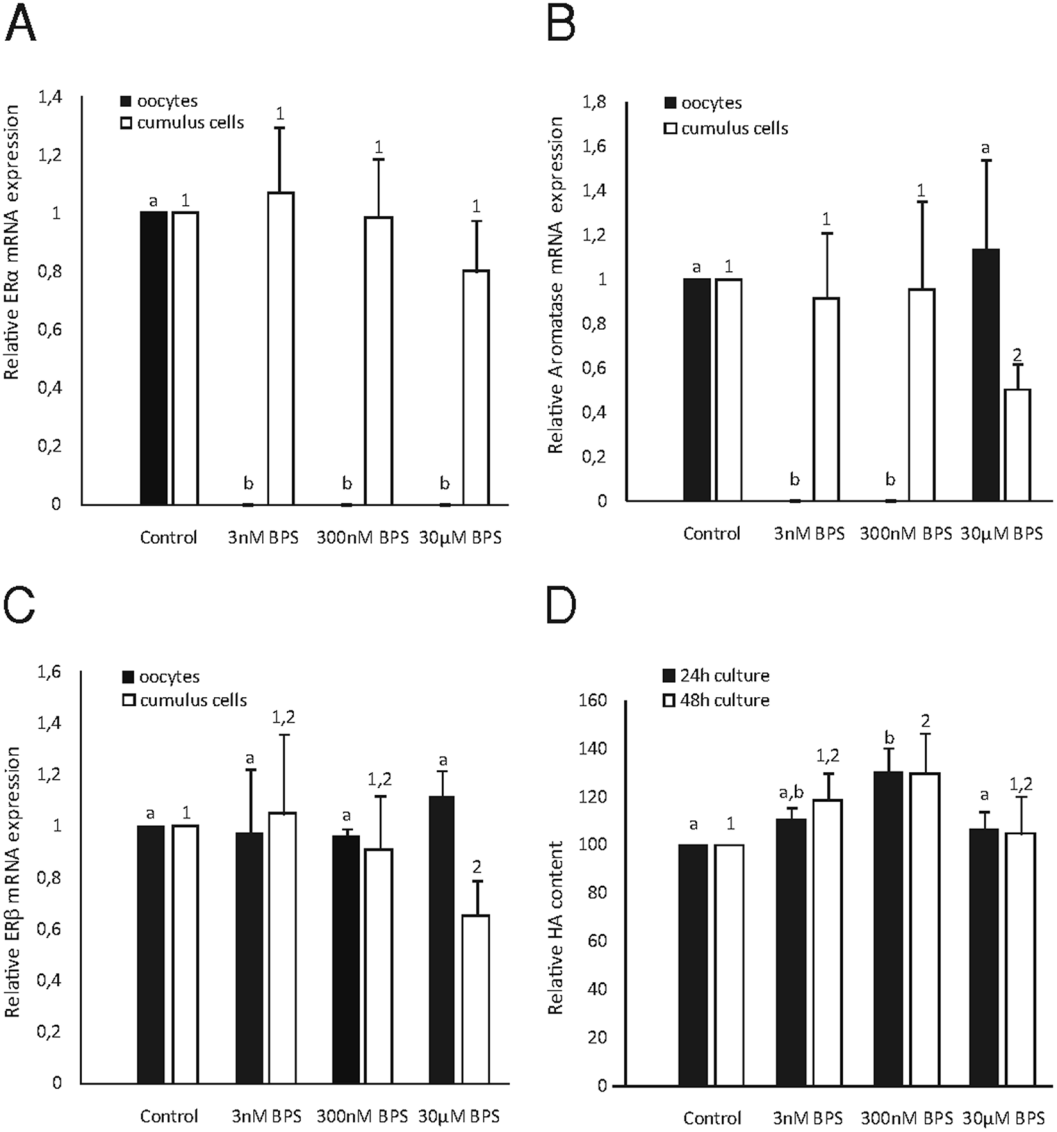
Effects of BPS on mRNA expression levels of selected genes and cumulus cell expansion. Effects of BPS (3 nM, 300 nM, and 30 μM) on the relative mRNA expression of (**A**) ERα, (**B**) ERβ, and (**C**) aromatase in oocytes and cumulus cells cultured for 48 h *in vitro*. The data are expressed from three independent experiments, with a total of n = 150 oocytes per group. Different letters and numbers denote the statistical significance among experimental groups for oocytes and cumulus cells, respectively (P < 0.05). (**D**) Effects of BPS on HA content in COCs after 24 and 48 h of *in vitro* culture. Different letters and numbers denote the statistical significance among experimental groups for 24 and 48 h of *in vitro* culture, respectively (P < 0.05).

The oocyte is transcriptionally inactive during meiotic maturation; therefore, correct meiotic maturation is completely dependent on maternal reserves of gene transcripts. Important targets of the oestrogenic effects of BPS are the mRNA transcripts for ERα, ERβ, and aromatase. Our results indicated the presence of mRNA transcripts for ERα, ERβ, and aromatase in oocytes and cumulus cells, whose responses to BPS treatment differed based on transcript amounts. Notably, the amount of ERα transcripts in oocytes was dramatically decreased after BPS treatment regardless of concentration. Moreover, the amount of aromatase transcripts was dramatically decreased in oocytes treated with BPS concentrations of 3 nM or 300 nM. No changes in the amount of ERβ transcripts were observed in oocytes. In the cumulus cells surrounding the oocytes, mRNAs of aromatase and ERβ decreased after 30 µM BPS treatment (Fig. 3A–C).

### Effects of BPS on the expression and redistribution of ERα, ERβ, and aromatase during the maturation of porcine oocytes

**Figure 4:**
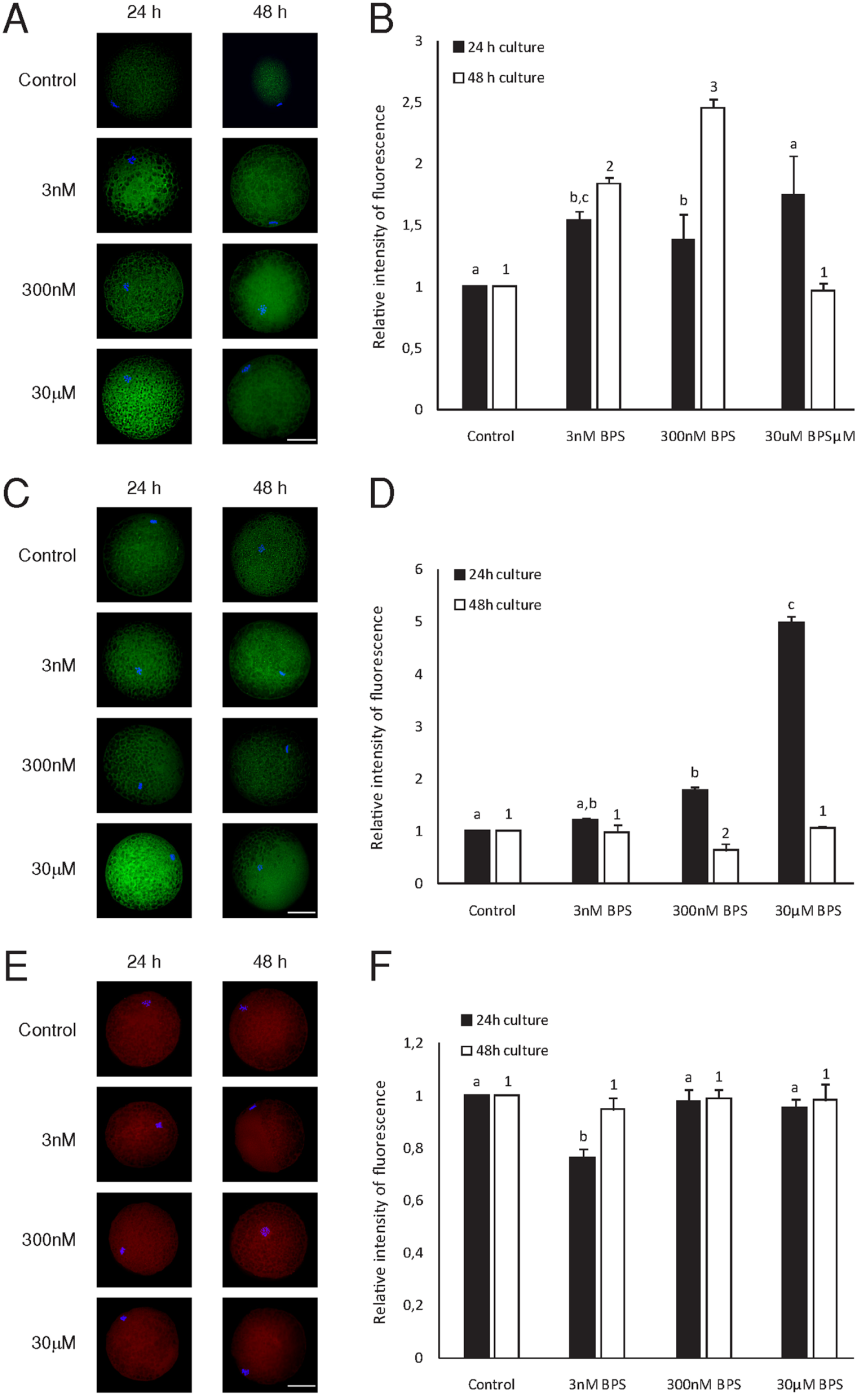
Effects of BPS on ERα, ERα, and aromatase during oocyte maturation. Representative pictures showing changes in the distribution of ERα (**A**), ERβ (**C**), and aromatase (**E**) in oocytes cultured for 24 h and 48 h *in vitro* after BPS (3 nM, 300 nM, and 30 µM) treatment. Green colour indicates ERα and ERβ, red indicates aromatase, and blue indicates chromatin. Scale bar = 50 µM. Graphs (**B**), (**D**), and (**F**) represent differences in the relative fluorescence intensities of ERα, ERβ, and aromatase. The data are expressed as the mean ± SEM of three independent experiments in which at least 20 oocytes were analysed. Different superscript letters denote the statistical significance among experimental groups for 24 and 48 h of *in vitro* culture, respectively (P < 0.05).

The presence of ERα, ERβ, and aromatase was observed throughout the entire meiotic maturation process. The expression and distribution of ERα and ERβ were significantly altered during *in vitro* culture. Notably, these changes were detected during the first meiotic division in which treatment with 30 µM BPS significantly increased the signal intensity of ERα and ERβ. Moreover, the 300 nM BPS treatment also affected these two factors in MI and MII oocytes. Differences were also observed in aromatase expression and distribution within MI oocytes treated with 3 nM BPS (Fig. 4A–F).

### Changes in HA-derived cumulus expansion after BPS treatment

During oocyte maturation *in vitro*, cumulus cells produce large amounts of extracellular matrix in which HA is the most abundant compound. In the presence of BPS, HA production in cumulus cells significantly changed. After 300 nM BPS treatment, HA production increased after 24 and 48 h of *in vitro* culture. Interestingly, the other tested BPS concentrations (3 nM and 30 µM) did not influence HA production in COCs (Fig. 3D).

## Discussion

To the best of our knowledge, this is the first study to investigate the relationship between BPS exposure and the maturation of mammalian oocytes. Our results demonstrate the markedly negative impact of BPS on pig oocyte maturation *in vitro*, specifically in terms of cell cycle blockade, cytoskeletal disruption, changes in the mRNA levels of key BPS targets, and changes in cumulus expansion. The negative effects of BPS on pig oocyte maturation were also apparent at concentrations that were orders of magnitude lower than BPS concentrations observed in human blood serum and urine^17^. Pig oocyte physiology shares many similarities with that of human oocytes. Importantly, pig oocytes have high sensitivity to the negative effects of BPA during *in vitro* maturation^24^. Therefore, our results are reliably applicable to human reproduction.

Because oestrogens are highly prevalent in the environment, it was necessary to exclude background BPS in our experiments to evaluate the effects of low doses of BPS. Environmental factors altering the composition of follicular fluid harm oocyte competence, either via direct effects on the oocyte itself or by indirectly affecting follicular cells or hormonal actions. Therefore, knowledge of the history of oocytes placed into an *in vitro* maturation system is required^25^. The follicular fluid creating the microenvironment for our porcine oocytes was analytically demonstrated to be free of BPS. The oocytes used in our experiments were exposed only to the BPS concentrations that were added into the culture medium (see Supplementary Tables S1 and S2). In addition to this fact, BPS doses used in our experiments respect concentrations measured in human blood serum and urine (0.8–84 nM)^15, 16^.

The effects of BPS on the course of the meiotic cell cycle were evaluated at different time intervals during *in vitro* oocyte maturation. Under *in vitro* conditions, BPS blocked the maturation of some oocytes in MI and/or at the exit from MI. After 24 h of culture, oocytes reached the MI stage with decreased success, whereas all oocytes reached the MI stage after 48 h of maturation, but a portion did not continue in meiosis up to MII. These effects were dose-dependent. Given the observed effects of BPS on pig oocyte maturation, the period around MI appears to be critical. This phenomenon was also demonstrated by our experiments in which BPS exerted substantial effects on *in vitro* oocyte maturation during the first 24 h period and the second 24 h period (see Supplementary Fig. S1). However, it was only possible to determine whether these effects were attributable to a slowing of the cell cycle or meiotic maturation blockade after culture was prolonged beyond 48 h. Significantly, a proportion of oocytes exposed to BPS remained in the MI or anaphase I/telophase I stages. Therefore, BPS not only causes a slowing of pig oocyte maturation *in vitro*, similar to that observed during the maturation of mouse oocytes in the presence of BPA^26^ or bisphenol AF^27^, but BPS also permanently blocks the course of maturation in a significant portion of oocytes. Similar effects have been observed for the maturation of pig oocytes in BPA presence^24^. Although the effects of low doses of endocrine disruptors are not surprising^28^, we demonstrated significantly negative effects with a very low dose of BPS (3 nM), which has not previously been observed in experiments investigating the effects of BPA on mammalian oocyte maturation.

Oocyte sensitivity to BPS during the period surrounding MI appears to be related to meiotic spindle formation. In our experiments, even oocytes cultivated in only 3 nM BPS were distinctly damaged. Based on our results, BPS impairs meiotic spindle creation in pig oocytes and causes irregularities in the arrangement of tubulin fibres. Similar effects on chromosome congression failure *in vivo* ^29^ and *in vitro* ^26, 30^ were observed during mouse oocyte maturation in the presence of BPA; meiotic spindle abnormalities apparently resulted from spindle checkpoint control failure. During the *in vitro* maturation of pig oocytes, BPA exerted negative effects on cell cycle progression, spindle architecture, and chromosome organization^24^. These effects may be attributable to the influence of BPS on oestrogens: oestrogens, specifically oestradiol, affect the regulation of mammalian oocyte maturation *in vitro* ^31, 32^, and increased concentrations result in meiotic spindle defects^32^. In our experiments, individual meiotic spindle defect frequencies were not linearly dependent on dosage, as is often the case with endocrine disruptors^33–35^. A similar non-linear effect was observed in terms of the effects of BPA on human oocyte maturation *in vitro* ^36^. In our study, we demonstrated for the first time the impact of BPS on cytoskeletal structures and noted equally dangerous effects compared to those confirmed in studies investigating BPA.

Our results demonstrate the presence of mRNA transcripts for frequent targets of endocrine disruptors, specifically ERα, ERβ, and aromatase, both in oocytes and in cumulus cells. After *in vitro* culture with BPS at low concentrations, mRNA transcripts for ERα and aromatase were no longer detectable in oocytes. This phenomenon was related to the non-linear effects induced by the endocrinologically disruptive actions of BPS. The ability of BPS to regulate mRNA expression was previously confirmed only in somatic cells^37^. BPA alters the global supply of gene transcripts connected to key cell processes in oocytes^38^. Altered signalling during processes leading to destabilization of the overall maternal stock of mRNA in oocytes^39^ or its selective degradation^40^ may be responsible for the decrease in mRNA transcript levels that we observed. This phenomenon might also be explained by high levels of translation and the required presence of proteins to sustain or release from the first meiotic block^41^. Somatic cumulus cells play a role in transferring transcripts into the oocyte and also enlarge maternal mRNA stocks within the oocyte^42^. Our results also demonstrate the decreased expression of mRNA transcripts for ERβ and aromatase in cumulus cells surrounding oocytes exposed to 30 µM BPS, which may result in the decreased transport of these mRNAs from the cumulus cells into the oocyte. These effects are potentially attributable to toxicity, which would be in accordance with the effects of BPA described in somatic cell lines^43^. The different effects of BPS on ERα and ERβ transcripts may be related to the affinity of ERs to BPS^44^. Thus, BPS may trigger diverse translation responses. The decrease in ERα transcripts may be explained by a nonlinear relationship between the number of bound receptors and the strongest observable biological effect^35^. Similarly, aromatase transcript expression has also shown a non-monotonic effect, in which low doses appear to be more effective than high doses in altering transcript levels. Decreases in the amount of transcripts can be explained by increased translation as well as disruption of transcript stability (*e*.*g*., due to polyadenylation of mRNAs)^18, 45^. BPS may thus affect both of these mechanisms of transcription regulation. In general, the same concentrations of BPS may exert even more damaging impacts on oocytes than on cumulus cells in terms of decreasing mRNA transcript levels, suggesting female gametes are more sensitive than somatic cells to the endocrinologically disruptive actions of BPS. At the same time, there may be different BPS signalling mechanisms in somatic cells versus oocytes.

Our results indicate direct ERα and ERβ protein expression in oocytes. Moreover, BPS also possesses the ability to influence meiotic maturation by targeting oestrogen receptors. Culture with BPS disrupts the expression of ERα and ERβ, as seen in MI and MII. In somatic cells, BPS acts as a weak agonist of oestrogen receptors^46^ present in a number of tissue types. During somatic cell mitosis, ERα regulates chromosome alignment and spindle dynamics by stabilizing microtubules during metaphase^47^. The absence of ERα mRNA in mature oocytes may be associated with increased ERα signal intensity during *in vitro* maturation, thus suggesting transcript depletion during ERα translation. Moreover, BPS-induced alterations in ERα signal intensity after 24 and 48 hr of *in vitro* culture may affect microtubule function, thus causing the spindle malformations^47^ observed in our experiments. Both oestrogen receptors clearly increased after 24 hr of *in vitro* culture with BPS. Although the amount of ERα protein was accompanied by decreases in mRNA, ERβ mRNA was not affected. This observation suggests that the ubiquitin-proteasome system^48^ may be targeted when proteolytic degradation of ERβ is protracted. However, the amount of ERβ protein did not increase after 48 hr of *in vitro* culture; in contrast, stimulation of proteolytic degradation appeared to occur after 300 nM BPS treatment, in a manner potentially promoted by receptor saturation^49, 50^. Noticeably, BPS, simulating oestrogen action, affect dynamics of oestrogen receptors due to both post-transcriptional and post-translational regulation^50, 51^.

In addition to endocrine disruptors, the expression of aromatase, which is responsible for steroidogenesis, is affected in porcine oocytes. Therefore, cross-talk between aromatase-derived oestrogens and endocrine disruptors is a target of BPS in porcine oocytes, and our evidence points to endocrinological disruption by BPS, which affects mammalian oocyte maturation *in vitro*. According to our results, BPS increases the levels of retained HA in cumulus–oocyte complexes. This effect was observed after 24 h and 48 h of *in vitro* culture at concentration of 300 nM BPS. BPA also alters HA levels by decreasing the amounts of retained HA^22^ and suppressing the cumulus expansion of pig COCs^24^. Presumably, 300 nM BPS may mimic hormonal stimulation of cumulus expansion within 24 h cultured oocytes. Futhermore, BPS can also affect paracrine regulation factors (*i*.*e*., insulin-like growth factor and growth differentiation factor-9)^52, 53^ and other key molecules, such as hyaluronan synthase-2, cAMP, and/or microRNAs^54, 55^. The proposed BPS sensitivity of cumulus expansion regulatory mechanisms is consistent with earlier observations on the effects of other endocrine disruptors on cumulus-oocyte complexes and cumulus expansion^22^. BPS exerts different effects as oocyte maturation progresses. The non-linear effect of BPS is apparent when 3 nM and 30 µM BPS treatments do not show significant effects. This differing mechanism may also underlie the effects on mRNA expression observed at the low doses evaluated in our study. Furthermore, our findings correspond to the aforementioned non-linear effects of BPS.

In conclusion, based on the results of our study, mammalian oocytes are highly sensitive to the effects of BPS. This is the first study describing the impact of low doses of BPS on mammals. The presented results help to clarify the mechanism by which endocrine disruptors influence mammalian reproduction and suggest that the ever-increasing use of BPS does not constitute a safer alternative to BPA.

## Methods

### Reagents

Chemicals were obtained from Sigma-Aldrich (St. Louis, MO, USA) unless otherwise noted.

### Porcine oocyte isolation and culture

The authors declare that the present study was carried out in accordance with the current laws of the Czech Republic and all the experimental protocols were approved by the Ethics Committee at the Czech University of Life Sciences Prague. Porcine ovaries were collected from pre-pubertal gilts at a local slaughterhouse. COCs were aspirated from medium-sized follicles using a 20-gauge needle. Only oocytes surrounded by several layers of cumulus cells and uniform ooplasm were selected for further study. Oocytes were cultured in M199 medium supplemented with sodium bicarbonate (0.039 mL of a 7.0% solution per 1 mL of medium), calcium lactate (0.6 mg/mL), gentamicin (0.025 mg/mL), HEPES (1.5 mg/mL), 13.5 IU of eCG plus 6.6 IU of hCG/mL (P.G. 600 Intervet, Boxmeer, Netherlands), and 5% foetal calf serum. Based on our preliminary experiments (data not shown), COCs were treated with BPS in following concentrations: 30 pM, 3 nM, 300 nM, and 30 μM, dissolved in DMSO to its final concentration of 0.1%. Vehicle control when COCs cultivated in medium with equal DMSO concentration was used. The oocytes were cultured for 24, 48, or 72 h in 5.0% CO_2_ at 39 °C.

### Oocyte evaluation

After culture, the oocytes were denuded from surrounding cumulus cells by pipetting. Thereafter, oocytes were mounted onto slides, fixed with acetic alcohol (1:3, v/v) for at least 24 h, and stained with 1.0% orcein. The oocytes were examined under a phase-contrast microscope (magnification 400x). The stages of oocyte nuclear maturation, specifically GV, late diakinesis (LD), MI, anaphase I (AI), telophase I (TI), and MII, were evaluated in accordance with previously described criteria^56^.

### Trypan blue staining of oocytes and cumulus cells

After 24 or 48 hr of *in vitro* cultivation, COCs (15 per group) were incubated with a 0.2 (w/v) solution of Trypan blue for 10 min. After incubation, oocytes were denuded, and Trypan blue-positive cells were counted. Cumulus cells were washed three times in PBS, and Trypan blue-positive cells were counted with a Thoma chamber when one hundred cells were evaluated.

### Oocyte immunofluorescence and imaging

After culture, oocytes were treated with 0.5% pronase to remove the *zona pellucida* and further processed as previously described^57^ with slight modifications. Oocytes were permeabilised and blocked (in 0.1% Triton X-100 dissolved in PBS supplemented with 1% and 5% normal goat serum, respectively), and then incubated overnight with the following antibodies (1:200; at 4 °C): anti-α-tubulin (T6199, Sigma-Aldrich), anti-CYP19/aromatase (LS-C188219, LifeSpan BioSciences, Seattle, WA, USA), anti-oestrogen receptor α (ab3575, Abcam, Cambridge, UK), and anti-oestrogen receptor β (ab3576, Abcam). Subsequently, oocytes were washed twice before incubation with fluorescein isothiocyanate (FITC)-conjugated goat anti-mouse IgG, FITC-conjugated goat anti-rabbit IgG, and tetramethylrhodamine isothiocyanate (TRITC)-conjugated goat anti-mouse IgG (Thermo Fisher Scientific, Waltham, MA, USA; 1:200). Thereafter, oocytes were washed twice and mounted in Vectashield containing 4′6-diamidino-2-phenylindole (DAPI; Thermo Fisher Scientific). Images were acquired using a Ti-U microscope (Nikon Co., Tokyo, Japan) to detect ERα/ERβ and aromatase. A confocal scanning microscope (Leica, SPE, Germany) was used for α-tubulin visualization. Exposition conditions were the same for each individual protein, and its negative control, which lacked a specific antibody, was processed under comparable conditions. Image analysis was performed using NIS Elements (Laboratory Imaging Ltd, Prague, Czech Republic). Aromatase, ERα, and ERβ signal intensities were normalized to the basal signal intensity of the negative control and compared to those in untreated oocytes.

### Western blot analysis

Samples were prepared in accordance with previous study^58^. Briefly, denuded GV oocytes (200 per sample) were placed into 15 µL of sample buffer. Surrounding cumulus cells were processed separately. Samples were heated at 100 °C for 5 min and proteins were separated using 12.5% SDS-PAGE and then electrophoretically transferred to a nitrocellulose membrane (GE Healthcare, Amersham UK). A pre-stained molecular weight standard (Bio-Rad Laboratories, Waltford, UK) was used to verify the molecular weights of the detected proteins. After overnight blocking in 2% milk in phosphate-buffered saline (PBS) containing 0.1% Tween 20, the membrane was incubated for 2 h with primary antibodies at a concentration of 1:500 for β-Actin (#4970, Cell Signaling Technology, Davers, MA, USA; as an internal loading standard), ERα and ERβ, and 1:250 for aromatase. The membrane was incubated with a secondary mouse or rabbit IgG antibody (GE Healthcare) at a concentration of 1:10,000 or 1:40,000, respectively. The proteins were visualized using an ECL Select Western Blotting Detection Kit (GE Healthcare) and a C-Digit Blot Scanner (LI-COR Biosciences, Lincoln, NE, USA).

### mRNA analysis

Samples for the quantitative real-time polymerase chain reaction (RT-qPCR) analysis of aromatase, ERα, and ERβ mRNAs were prepared from immature GV and mature MII oocytes (50 oocytes in each group). Concurrently, cumulus cells were employed for the same analysis. RNA was isolated using a 6100 Nucleic Acid PrepStation (Fisher Scientific, USA) in accordance with the instruction manual. Total mRNA was transcribed to cDNA using a High-Capacity cDNA Achieve Kit (Thermo Fisher Scientific) in accordance with the manufacturer’s instructions. cDNA was synthesized in a final volume of 100 μL. Sets of specific primers were synthesized in accordance with known sequences to amplify specific products for GAPDH, aromatase, ERα, and ERβ (see Supplementary Table S4). Each PCR reaction was performed in triplicate in a total volume of 10 μL with the gene-specific primers at 500 nM and the TaqMan MGB probe at 200 nM, 5 μL of 2x concentrated Fast TaqMan Universal Master Mix (Thermo Fisher Scientific), 1 μL of cDNA, and nuclease-free water up to a volume of 1 mL. The 7500 Fast Real-Time PCR System (Thermo Fisher Scientific) was utilized for RT-qPCR reactions with the following programme: 95 °C for 20 s followed by 40 cycles of 95 °C for 2 s and 60 °C for 20 s. mRNA expression was quantified for each enzyme using SDS software and the arithmetic formula 2^−ΔΔCT^ in accordance with the comparative Ct method^59^ to determine the amount of the target normalized to the GAPDH endogenous control as a reference^45^, relative to fully grown GV oocytes and their cumulus cells.

### HA measurement and the evaluation of cumulus expansion

Groups of 25 COCs were cultured for 24 or 48 h as described above and processed as previously described^58, 60^. Briefly, the COCs were three-times washed in PBS-PVA, and oocytes were mechanically denuded by repeated pipetting. Isolated HA was enzymatically digested with lyase from *Streptomyces hyalurolyticus* (2 IU/mL) at 39 °C overnight. Subsequently, HA solutions were spectrophotometrically measured using a Helios Gamma spectrophotometer (Thermo Fisher Scientific) at 216 nm against a blank consisting of PBS-PVA containing lyase. The quadratic calibration curve was based on five HA standards (0.006–0.1% sodium hyaluronate) digested via the protocol used for the samples. The concentration of HA was expressed as the retained HA relative to the untreated control group.

### LC-MS/MS analysis of BPS in porcine follicular fluid

Follicular fluid samples were prepared during oocyte aspiration in accordance with the previously described oocyte collection. The follicular fluid was obtained from three independent aspirating sessions. Subsequently, a sample preparation method described by^61^ was employed with modifications. Briefly, samples were centrifuged, and 2 mL of supernatant was added to 1 mL of 200 mM sodium acetate buffer (pH 5.4) together with a (^13^C_12_) internal standard (10 µL of a 50 ng/mL solution), followed by incubation with 20 µL of beta-glucuronidase/arylsulfatase from *Helix pomatia* (Roche, Mannheim, Germany) for 5 h at 37 °C. Samples were extracted with 2 mL of acetonitrile and 3 mL of ethyl acetate. After sonication (40 kHz for 10 min.) and centrifugation, 4 mL of supernatant was evaporated under nitrogen at 60 °C, and the residue was reconstituted with 0.5 mL of 50% methanol in water. Samples were analysed on a Dionex Ultimate 3000 UHPLC system (Thermo Fisher Scientific) coupled to a 3200 QTRAP triple quadrupole mass spectrometer (AB Sciex, DC). The following liquid chromatography conditions were used: Phenomenex Kinetex C18 column (30 × 2.1 mm, 1.7 µm), column temperature 35 °C, autosampler temperature 10 °C, flow 0.3 mL/min, injection volume 10 µL. Mobile phase (A) was methanol, and phase (B) was water. The following gradient was employed: 0 min 90% B, 0.2 min 90% B, 4.5 min 10% B, 5.5 min 10% B, 6.5 min 90% B, and 8 min 90% B. The following mass spectrometry parameters were used: the ESI source was operated in negative mode at 600 °C, ion spray voltage −3500 V, curtain gas 20 a.u., nebulizer gas 35 a.u., turbo gas 25 a.u., collision gas “medium”, ion dwell time 70 ms, ions registered: 249.1/107.9/155.9/92.0 for bisphenol S and 261.1/114.1/98.1/162.1 for the internal standard. The BPS retention time was approximately 2.55 min. Eight-point linear calibration (r = 0.9999) ranged from 0.05 ng/mL to 100 ng/mL.

### Statistical analysis

The data are presented as the mean ± SEM of at least three independent experiments. The general linear models (GLM) procedure, following the Shapiro-Wilk test of normality, was employed in SAS package 9.3 (SAS Institute Inc., Cary, NC, USA) to analyse data from all experiments. Significant differences among groups were determined using Sheffé’s test. P < 0.05 was regarded as statistically significant.

## Electronic supplementary material

Video 1 - tubulin and chromosomes

Supplementary Information
