# Supplementary material for: Bisphenol S negatively affects the meotic maturation of pig oocytes
Source: Sci Rep. 2017 Mar 28;7:485. doi: 10.1038/s41598-017-00570-5 (PMC5428703; doi:10.1038/s41598-017-00570-5)
Supplement: Supplementary file 2 — Supplementary Information [file 41598_2017_570_MOESM2_ESM.pdf]

Bisphenol S negatively affects the meotic maturation of pig oocytes

Tereza Žalmanová[1*], Kristýna Hošková[1], Jan Nevoral[1,2], Kateřina Adámková[1], Tomáš Kott[3], Miloslav Šulc[4], Zora Kotíková[4], Šárka Prokešová[1], František Jílek[1], Milena Králíčková[2], and Jaroslav Petr[3]

[1]*Czech University of Life Sciences Prague, Faculty of Agrobiology, Food and Natural Resources, Department of Veterinary Sciences, Prague, Czech Republic*
[2]*Charles University in Prague, Faculty of Medicine in Pilsen, Biomedical Center and Department of Histology and Embryology, Pilsen, Czech Republic*
[3]*Institute of Animal Science, Prague, Czech Republic*
[4]*Czech University of Life Sciences Prague, Faculty of Agrobiology, Food and Natural Resources, Department of Chemistry, Prague, Czech Republic*

[*]**Correspondence to:** Tereza Žalmanová, Czech University of Life Sciences Prague, Faculty of Agrobiology, Food and Natural Resources, Department of Veterinary Sciences, Prague, Czech Republic. Tel: 00420 224382942. E-mail:zalmanovat@af.czu.cz

Supplementary information

Supplementary Table S1 and Table S2: Analytical assessment of bisphenol S (BPS) in follicular fluid.

Our LC-MC/MC analysis have shown no observable presence of BPS in porcine follicular fluid, creating microenvironment of ovarian follicles where oocytes are enclosed. All measured values of BPS in follicular fluid samples lied under the quantitation limit (LOQ; <0.125 ng/mL), which in accordance with results of verification experiments performed on various body fluids (Table S1). Data from measured samples originated from three independent follicular fluid aspirating session are shown in Table S2. Our findings support that the obtained COCs were not under significant BPS influence during in vivo oogenesis.

Table S1. Basal output of introduced LC-MC/MC analysis of BPS.

sample	Limit of quantitation [ng/mL]	Analyte recovery [%]	Intra-day Repeatability* [%]	Inter-day Repeatability** [%]
BPS standard	0.05	95	6.3	8.2
Follicular fluid	0.125	55	3.8	5.6
Oviductal fluid	0.125	60	5.1	7.8
Urine	0.250	62	11.4	12.9

* average of 10 independent analyses
** average over 7 days (blank matrix samples spiked with 3 µg/mL BPS)

Table S2. The output of LC-MS/MS analysis of BPS content in porcine follicular fluid.

Sample No.	Date of collection	Measured value (ng/mL)
#1	April 24	<LOQ
#2	April 24	<LOQ
#3	April 24	<LOQ
#4	April 24	<LOQ
#5	April 29	<LOQ
#6	April 29	<LOQ
#7	April 29	<LOQ
#8	April 29	<LOQ
#9	May 19	<LOQ
#10	May 19	<LOQ
#11	May 19	<LOQ
#12	May 19	<LOQ

Supplementary Table S3: Trypan blue staining for assessment of viability of oocytes (A) and their cumulus cells (B).

A)

Trypan blue positive cells (%)	24h culture	48h culture
Control group	2.2 ± 0.04	2.2 ± 0.04
3 nM BPS	2.2 ± 0.04	4.4 ± 0.04
300 nM BPS	2.2 ± 0.04	2.2 ± 0.04
30 μM BPS	4.4 ± 0.04	4.4 ± 0.04

B)

Trypan blue positive cells (%)	24h culture	48h culture
Control group	5.7 ± 0.01	8.3 ± 0,01
3 nM BPS	7.3 ± 0.01	9.3 ± 0.01
300 nM BPS	6.7 ± 0.01	8.3 ± 0.01
30 μM BPS	7.3 ± 0.02	9.3 ± 0.02

Effects of BPS (3nM, 300nM, and 30μM) on the viability of oocytes (A) and cumulus cells (B) after 24h and 48h *in vitro* culture. The trypan blue dye exclusion method was used for evaluation of viability of the cells. The data are expressed as the mean ± SEM (45 oocytes and 300 cumulus cells inthree independent experiments). Our results indicate only basal level of cell death (*i.e.* Trypan blue positive cells), regardless BPS treatment. Accordingly, no significant differences ($P < 0.05$) among groups were observed.

Supplementary Figure S1: Effects of BPS on the meiotic maturation of oocytes.

A)

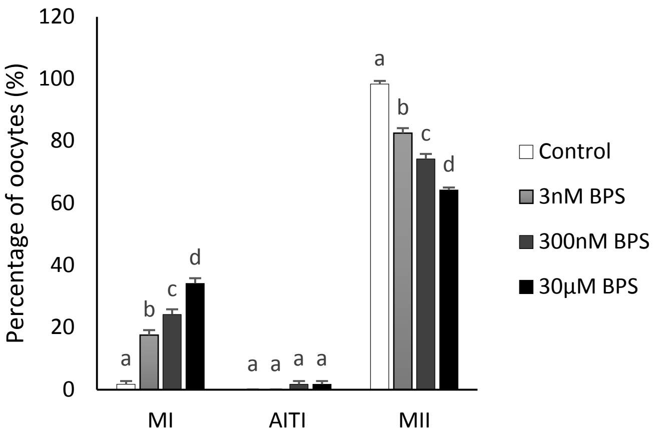

B)

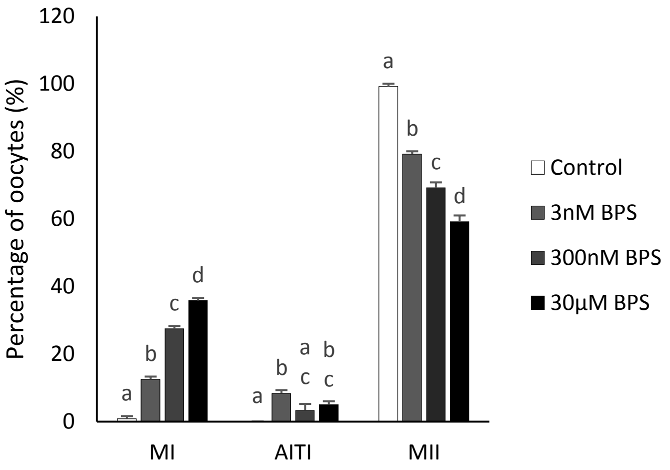

C)

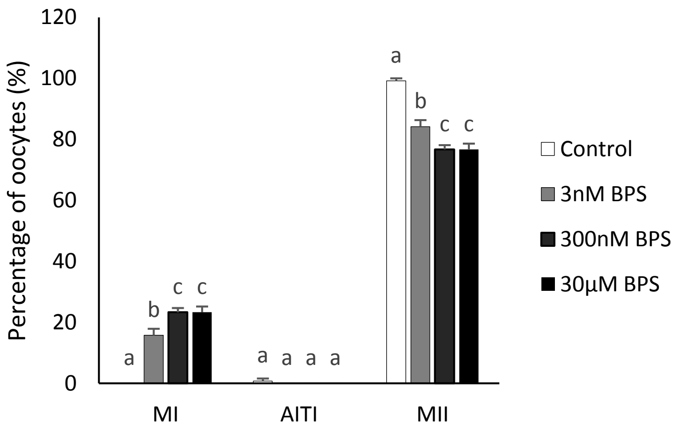

Effects of BPS (3 nM, 300 nM, and 30 µM) on the stages of meiotic maturation achieved by oocytes cultured A) after 48 h of culture with BPS followed by 24 h culture in a BPS-free medium, B) after 24 h of culture with BPS followed by 24h culture in a BPS-free medium, C) after 24 h of culture with BPS followed by 24 h culture in a BPS-free medium *in vitro*. MI – metaphase I, AITI – anaphase I–telophase I, MII – metaphase II. The data are expressed as the mean ± SEM from four independent experiments, n = 120 oocytes per group. Different superscripts denote statistical significance ($P < 0.05$).

Supplementary Figure S2: Test of specificity of used antibodies.

Evaluation of specificity of used antibodies A) ERα, B) ERβ, and C) aromatase in GV oocytes and their cumulus cells by western blotting. Anti-β-Actin antibody was used as an internal loading standard.

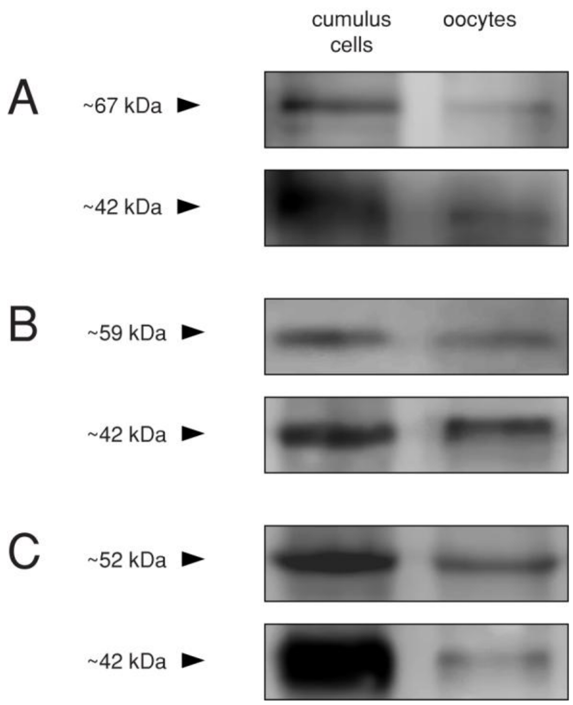

Supplementary Video 1: Effect of BPS on α-tubulin organisation in the oocyte.

Oocyte after 48h *in vitro* culture with 300 nM BPS. Individual images from the Z-stacks were used to 3D projection of chromosomes and α-tubulin assessment. Z-stack projections were generated in LAS AF software. Scale bar = 10 µM.

Supplementary Table S4: Specific primers and probes used for mRNA analysis.

Gene	Accession number	Forward primer (5'→ 3')	Reverse primer (5'→ 3')	MGB probe (5'→ 3')	Product size (bp)
**GAPDH**	AK234838.1	GAGCATCTCCTGACTTCCAGTTTC	CCTAAGCCCCTCCCCTTCT	ATCCCAGACCCCC	60
**ER α**	AF035775	GGACAGGAACCAGGGCAAGT	AGCCAGCAACATGTCAAAGATCT	TGTCGAGGGAATGGT	61
**ER β**	AF267736	ATGTGGCGCTCCATCGA	CAGAACGAGGTCTGGAGCAAA	CCGGCAAGCTCAT	57
**Aromatase**	L15471.1	CTGTCGTGGACCTCGTCATG	TCACCGGGTAGCCATCGAT	AAAAGCCTTAGAGGATGATG	63
